# Vascular and Alzheimer's disease markers independently predict brain atrophy rate in Alzheimer's Disease Neuroimaging Initiative controls

**DOI:** 10.1016/j.neurobiolaging.2013.02.003

**Published:** 2013-08

**Authors:** Josephine Barnes, Owen T. Carmichael, Kelvin K. Leung, Christopher Schwarz, Gerard R. Ridgway, Jonathan W. Bartlett, Ian B. Malone, Jonathan M. Schott, Martin N. Rossor, Geert Jan Biessels, Charlie DeCarli, Nick C. Fox, Alzheimer's Disease Neuroimaging Initiative

**Affiliations:** a Dementia Research Centre, Department of Neurodegenerative Disease, University College London Institute of Neurology, London, UK; b Department of Neurology, University of California–Davis, Davis, CA, USA; c Wellcome Trust Centre for Neuroimaging, University College London Institute of Neurology, London, UK; d Department of Medical Statistics, London School of Hygiene and Tropical Medicine, London, UK; e Department of Neurology and Neurosurgery, University Medical Center Utrecht, the Netherlands

**Keywords:** Alzheimer's disease, Vascular disease, Mild cognitive impairment (MCI), Volumetric MRI, Normal aging

## Abstract

This study assessed relationships among white matter hyperintensities (WMH), cerebrospinal fluid (CSF), Alzheimer's disease (AD) pathology markers, and brain volume loss. Subjects included 197 controls, 331 individuals with mild cognitive impairment (MCI), and 146 individuals with AD with serial volumetric 1.5-T MRI. CSF Aβ1-42 (n = 351) and tau (n = 346) were measured. Brain volume change was quantified using the boundary shift integral (BSI). We assessed the association between baseline WMH volume and annualized BSI, adjusting for intracranial volume. We also performed multiple regression analyses in the CSF subset, assessing the relationships of WMH and Aβ1-42 and/or tau with BSI. WMH burden was positively associated with BSI in controls (*p* = 0.02) but not MCI or AD. In multivariable models, WMH (*p* = 0.003) and Aβ1-42 (*p* = 0.001) were independently associated with BSI in controls; in MCI Aβ1-42 (*p* < 0.001) and tau (*p* = 0.04) were associated with BSI. There was no evidence of independent effects of WMH or CSF measures on BSI in AD. These data support findings that vascular damage is associated with increased brain atrophy in the context of AD pathology in pre-dementia stages.

## 1 Introduction

Alzheimer's disease (AD) is the most common primary pathological cause of dementia, and vascular disease has been reported as the second most common (Brayne et al., 2009; Jellinger, 2006). Some studies have shown that AD and vascular pathologies are equally prevalent and that they can often co-occur (Brayne et al., 2009; Jellinger, 2006; Schneider et al., 2007; White et al., 2002). This is reflected in a recent statement to health care professionals to aid understanding of vascular contributions to cognitive impairment and dementia (Gorelick et al., 2011). It is unclear, however, how these differing pathologies have an impact on the disease progression from normal ageing to clinically manifest AD.

Longitudinal in vivo techniques to measure AD and vascular pathologies are now available to address this question. AD pathology can be examined in vivo by examining cerebrospinal fluid (CSF) levels of Aβ1-42, which are negatively associated with amyloid deposition in the brain (Shaw et al., 2009). CSF analysis also permits investigation of tau levels, which are thought to be positively associated with neuronal injury and brain atrophy (Jack et al., 2010). White matter hyperintensities (WMH) can be assessed in vivo using multi-spectral magnetic resonance imaging (MRI) acquisitions (Carmichael et al., 2010; Tiehuis et al., 2008). WMHs have multiple histopathological correlates, including ependymal loss, cerebral ischemia, demyelination, microcystic infarcts, venous collagenosis, and gliosis (Gouw et al., 2011; Kim et al., 2008). They increase with age and vascular risk factors (DeCarli et al., 2001; Jeerakathil et al., 2004; Nordahl et al., 2006; Yoshita et al., 2006). Furthermore, plasma amyloid levels are associated with WMHs, allowing for hypertension (Gurol et al., 2006). This association may represent a separate disease pathway from cerebral amyloid pathology, as plasma amyloid has not been shown to be associated with CSF amyloid levels (Le Bastard et al., 2010).

Brain atrophy is an important marker of disease progression in AD. Compared with normal ageing, rates of brain atrophy calculated using serial MRI scans are much higher in clinically diagnosed AD, with mild cognitive impairment (MCI) subjects showing intermediate rates (Henneman et al., 2009; Jack et al., 2004; Schott et al., 2005). These markers of disease progression are closely associated with cognitive decline (Evans et al., 2010).

Assessing the independent associations of CSF amyloid, CSF tau, and WMH with brain atrophy rate has the potential to identify contributions of vascular and AD pathologies to macroscopic brain loss over time. Understanding these relationships is especially important in a clinical trial setting in which imaging is used as an outcome measure. For example, more efficient trial designs may be achieved by adjusting or stratifying for vascular burden, or targeting those without significant vascular burden. Clinical trials of AD therapies are generally assumed to be largely free of cerebrovascular disease because they exclude individuals with clinically overt cardiovascular disease; in fact, WMHs may be prevalent and associated with greater cognitive decline in this setting (Carmichael et al., 2010). However. the independent impact of WMHs and AD markers on progressive brain atrophy in such clinical trial cohorts is not well understood.

The aim of this study was to investigate how baseline WMHs and CSF Aβ1-42 and tau relate to brain volume loss over the following year in controls and MCI and AD subjects enrolled in the Alzheimer's Disease Neuroimaging Initiative (ADNI), a multi-site natural history study.

## 2 Methods

We analyzed data from control, MCI, and AD subjects from the ADNI (adni.loni.ucla.edu) who had baseline and 1-year follow-up volumetric 1.5-T scans.

ADNI was launched in 2003 by the National Institute on Aging (NIA), the National Institute of Biomedical Imaging and Bioengineering (NIBIB), the Food and Drug Administration (FDA), private pharmaceutical companies, and non-profit organizations, as a $60 million, 5-year public–private partnership. The primary goal of ADNI has been to test whether serial MRI, positron emission tomography (PET), other biological markers, and clinical and neuropsychological assessment can be combined to measure the progression of MCI and early AD. Determination of sensitive and specific markers of very early AD progression is intended to aid researchers and clinicians to develop new treatments and to monitor their effectiveness, as well as lessen the time and cost of clinical trials.

The Principal Investigator of this initiative is Michael W. Weiner, MD, VA Medical Center and University of California—San Francisco. ADNI is the result of efforts of many co-investigators from a broad range of academic institutions and private corporations, and subjects have been recruited from over 50 sites across the United States and Canada. The initial goal of ADNI was to recruit the following: 800 adults, 55 to 90 years of age, to participate in the research; approximately 200 cognitively normal older individuals, to be followed up for 3 years; 400 individuals with MCI, to be followed up for 3 years; and 200 individuals with early AD, to be followed up for 2 years. (For up-to-date information, see www.adni-info.org).

Participants underwent baseline and periodic clinical and neuropsychometric assessments as well as serial MRI. Written informed consent was obtained, as approved by the Institutional Review Board at each participating center. Demographic, Mini-Mental State Examination (MMSE), genetic, CSF data, and cardiovascular risk factors were downloaded from the ADNI website (www.loni.ucla.edu/ADNI). Approximately 60% of participants had CSF taken for analysis (see: http://www.adni-info.org). Details of the analysis of the CSF for Aβ1-42 and tau have been described elsewhere (Shaw et al., 2009).

Baseline WMH volume was estimated from T1-, T2-, and proton density (PD)-weighted MR images using a previously described, automated technique (Carmichael et al., 2010; Schwarz et al., 2009). In brief, a linear combination of PD- and T2-weighted images were aligned to the T1-weighted image using rigid registration. MR images were stripped of non-brain tissues and non-linearly registered to a minimum deformation template. WMH were identified at each voxel in this template space, based on signal intensity of the voxel in all MR images, signal intensity of neighboring voxels, and prior probability of the existence of WMH. Brain volume at baseline was measured semi-automatically from T1-weighted images (Freeborough et al., 1997). Brain volume loss occurring between the serial T1 scans was quantified using an automated pipeline including brain segmentation (Leung et al., 2011) and the boundary shift integral (BSI), which gives an estimate of tissue loss over time directly from each scan pair (Freeborough and Fox, 1997; Leung et al., 2010b). Intracranial volume (TIV) was automatically estimated by summing the gray matter, white matter, and CSF segmentations using SPM8's new segmentation toolbox (http://www.fil.ion.ucl.ac.uk/spm/software/spm8). This toolbox uses prior probability maps for gray matter, white matter, CSF, bone, non-brain soft tissue, and air, improving the correspondence with manual measures compared with previous versions of SPM (Leung et al., 2010a; Ridgway et al., 2011).

Linear regression was used to estimate differences in means of continuous variables across diagnostic groups. For categorical variables, Fisher's exact test was used. Linear regression was used with annualized brain volume loss (based on BSI) as the dependent variable and combinations of WMH, CSF Aβ1-42, and tau as independent variables. Analyses were performed separately for each diagnostic group, with TIV included as a covariate. WMH burden was log-transformed (base 2) to reduce skewness. First we assessed the relationship of (log-transformed) WMH burden with annualized BSI, adjusting for head size. Because WMH was entered as a covariate after log (base 2) transformation, its estimated coefficient is the expected change in BSI corresponding to a doubling of WMH on the original scale. In a further analysis using the subset of subjects with baseline CSF available, we fitted the same regression model but with Aβ1-42 level included as a covariate. This analysis was repeated replacing Aβ1-42 with tau level. We further investigated a model with both CSF biomarkers included. For each variable we calculated the semi-partial r^2^ values to estimate the extent to which baseline WMH and CSF Aβ1-42 and tau independently explained subsequent brain volume loss. Furthermore, all analyses were repeated additionally adjusting for baseline brain volume to examine whether the established relationships could be explained by atrophy before the first scan. Finally we repeated the analyses adjusting for age to establish whether this could explain the associations of BSI with WMH and CSF markers. Scatter plots of annualized BSI against log_2_WMH with overlaid regression lines together with 95% CI were also generated to show unadjusted associations. Analyses were conducted in Stata 12.0.

## 3 Results

Table 1 shows demographic, *APOE,* and imaging summary statistics. Of note, subjects differed across diagnostic groups in terms of gender, with a higher proportion of males in the MCI group. As expected, subject groups also differed in terms of *APOE* ε4 dose, brain atrophy rate, WMH volume, and CSF tau levels, with these values increasing from controls to MCI to AD subjects. Groups also differed in terms of MMSE, CSF Aβ1-42, and brain volume/TIV, with these values decreasing from controls to AD subjects.

**Fig. 1:**
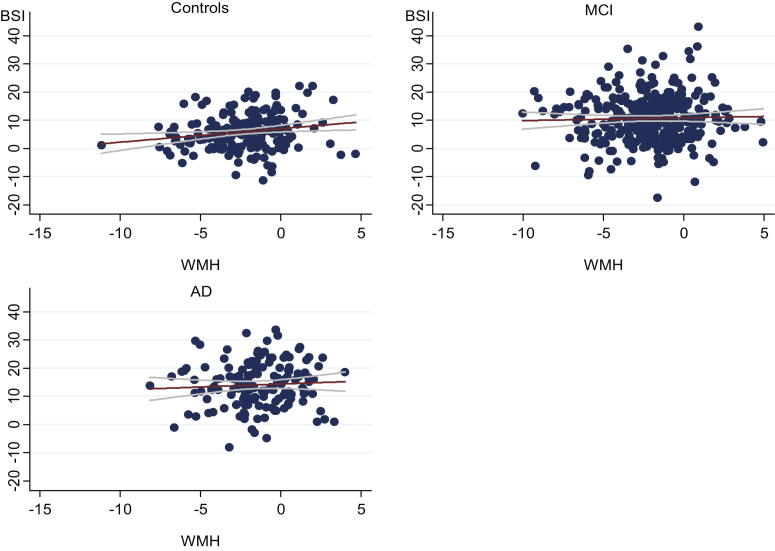
Scatter plots of annualized boundary shift integral (BSI [mL/y]) against white matter hyperintensities (WMH [log_2_mL]) in controls, subjects with mild cognitive impairment (MCI), and subjects with Alzheimer's disease (AD). Fitted regression lines (red) with 95% confidence interval for the predicted mean (gray).

Table 2 shows the partial regression coefficients for WMH, Aβ1-42, and tau, and Fig. 1 shows scatter plots of annualized BSI against log_2_WMH for each subject group. Without adjustment for CSF Aβ1-42 or tau, WMH burden was positively associated with brain volume loss in controls (after adjustment for head size). For the subjects with available baseline CSF, both WMH and CSF Aβ1-42 showed independent associations with volume loss in controls, with lower CSF Aβ1-42 and higher WMH volume associated with greater losses. Squared semi-partial correlations showed that, in control subjects, WMH explained an amount of variance in atrophy rates similar to that of Aβ1-42. There was no evidence of an independent association of tau with brain volume loss in controls. The WMH and Aβ1-42 results remained largely unchanged with adjustment for tau (model 4). The results in controls remained statistically significant after exclusion of the visible outlier, which can be seen in Fig. 1. The fitted regression equation for a control subject with a head size of 1500 mL is given by the following:

Mean volume loss (in mL) = 15.05 + (0.78 * log_2_WMH) + (−0.036 * Aβ1-42).

In MCI subjects, there was no evidence of an association between WMH and brain volume loss, either with or without adjustment for CSF Aβ1-42 or tau. There was evidence that CSF Aβ1-42 was associated with volume loss (independent of WMH, and independent of WMH and tau) in MCIs, and also evidence that increased tau was associated with increased loss. The association of tau and BSI no longer remained significant when additionally adjusted for Aβ1-42. In AD subjects, we found no evidence for effects of WMH, CSF Aβ1-42, or tau on brain atrophy rates.

All results remained largely unchanged when adjusting for baseline brain volume in addition to head size (see supplementary Table 1), suggesting that prior atrophy cannot explain the relationships found between WMH and CSF markers of AD pathology with subsequent brain volume losses. There was no evidence (*p* > 0.1, all tests) that brain volume was independently associated with BSI loss from these models, apart from model 3 (WMH and tau as covariates) in controls. Results from this model showed that a larger brain volume at baseline was associated with greater brain loss in the subsequent year (*p* = 0.046).

Results altered little when adjusting for age in addition to head size (see supplementary Table 2), suggesting that age does not explain the associations between atrophy rates with WMH and CSF Aβ 1-42 and/or tau. There was some evidence that age was independently negatively associated with BSI in the MCI (*p* < 0.05, all models) and AD (*p* < 0.05, models 1 and 3) groups, but not in controls (*p* > 0.6, all models).

There was no evidence of an effect of head size in any of the analyses reported above (*p* > 0.05, all tests).

## 4 Discussion

In this study, we found that both increased WMH volume and decreased CSF Aβ level were independently associated with an increase in brain volume loss (atrophy rate) in control subjects. In this subject group, WMH explained nearly as much variance in volume loss as CSF Aβ, with both explaining much more than tau. By contrast, in subjects with MCI or AD, in whom the atrophy rates were higher, WMH volume was not found to be associated with brain volume loss. Lower Aβ levels and higher tau were independently associated with higher volume loss in MCI patients; however, the tau association was no longer significant once adjusted for Aβ. Neither Aβ levels nor tau levels were found to be independently associated with brain volume loss in AD. Importantly, the results described are not materially altered by prior whole brain atrophy as represented by baseline brain volume and TIV, or age.

The finding that WMHs and Aβ levels are independently associated with longitudinal brain volume loss in individuals lacking clinically significant cognitive decline contributes to a growing body of literature suggesting that AD, vascular pathology, and mixed pathology are significant causes of neuronal loss accompanying ageing, even when this brain injury or neuronal loss has no clinically apparent cognitive manifestation. Importantly, this relationship was found among ADNI controls who were physically healthy, highly educated, of a high socioeconomic status, and were included only if overt cerebrovascular disease was not evident (all subjects had ≤4 Hachinski score points). Therefore, our findings are likely to under-represent the impact of WMH on brain ageing and therefore atrophy rates among members of the general population in whom Hachinski scores are likely to be higher (DeCarli et al., 2005). A previous pathological study has suggested that mixed pathology may be present in a high percentage of brains of normal individuals (White et al., 2002), and a subsequent in vivo study has suggested that additive contributions of AD and vascular disease to cognitive decline may be observable in normal subjects in their 80s (Wilson et al., 2010). The current study extends those findings by showing that independent effects of AD and vascular pathology on brain volume loss extend to very healthy elderly subjects.

The finding that only AD pathology, and not white matter pathology, was associated with brain atrophy within the MCI group may in part reflect the ADNI strategy of recruiting individuals whose amnestic pattern of MCI strongly suggested a predominance of AD pathology burden driving disease progression, as opposed to vascular disease. Although amnestic MCI subjects are at a high risk for conversion to AD, pathological studies of those recruited as amnestic MCI suggest that a significant proportion will not have underlying AD pathology (Jicha et al., 2006; Petersen et al., 2006). This means the MCI group is likely to represent a bimodal population of those with AD pathology and high rates of atrophy, as well as those with no pathology and low atrophy rates, thus further driving the association between amyloid levels and atrophy rates in this group. Proposed models of AD progression (Jack et al., 2010) suggest a strong relationship between tau and atrophy, especially in MCI. However published results from ADNI show mixed findings, with some studies demonstrating evidence of an association between baseline tau and atrophy rates (Fjell et al., 2010; Tosun et al., 2010) and others not finding such relationships (Leow et al., 2009; Schuff et al., 2009). These discrepancies, although potentially explained in part by the differing methodologies used, demonstrate that the relationship between baseline tau and subsequent atrophy is complex.

In AD subjects, the atrophy rate is much higher than in MCI subjects and controls, indicating that the disease is in a different stage with rapid progression. The fact that, in ADs, no evidence of associations were seen between atrophy rate and white matter pathology, Aβ, or tau, may reflect the fact that this population is more likely to have underlying AD pathology and is therefore more homogeneous (Jellinger, 2006), reducing power to detect associations. Previous studies have similarly found no evidence of an association between Aβ pathology and rate of atrophy in AD patients (Josephs et al., 2008; Sluimer et al., 2010), suggesting that Aβ load is a weak marker of severity or progression at this stage of the disease. Factors that may explain variance and drive brain volume loss at this disease stage are yet to be determined, but may include both genes and proteins involved in inflammatory responses and apoptosis, as well as disease duration.

Our finding of increased WMH being associated with longitudinal changes in brain volume is in keeping with other cross-sectional analyses of brain volume and WMH in normal subjects (Godin et al., 2009; Wen et al., 2006). Further, our study is in keeping with other longitudinal findings that have revealed that increased WMH volume at baseline was associated with greater changes in ventricular CSF in subjects who were cognitively intact at baseline (Silbert et al., 2008) and change in WMH was associated with change in brain volume in a large community based study (Debette et al., 2011). Our study adds to this literature by assessing the independent relationships between WMH and CSF Aβ1-42 and tau at baseline and brain volume losses over the following year in 3 diagnostic groups representing the range in clinical status from normal ageing to AD. Our study builds on an emerging model of the vascular contribution to decline in AD, which suggests that vascular damage is associated with cognitive decline in the context of AD predominantly in pre-dementia stages (Carmichael et al., 2012; Debette et al., 2011; Wilson et al., 2010). Furthermore, it may be that in the control group, some subjects have incipient AD, some have incipient vascular cognitive impairment, and some have incipient mixed vascular and AD. The relationship between BSI and potential explanatory variables is complex. Future work is required to elucidate which factors may be important and how these may interact (for example, age and white matter disease).

WMH volume results have been reported previously for the ADNI dataset (Carmichael et al., 2010), as have CSF results (Shaw et al., 2009). Atrophy rates have also been previously reported in different subsets of the ADNI dataset (Evans et al., 2009; Leung et al., 2010b; Schott et al., 2010a; Schott et al., 2010b).

This study has a number of limitations. First, we have no post mortem proof of clinical diagnosis, and therefore we cannot investigate whether a proportion of subjects in each diagnostic group had significant underlying vascular cognitive impairment. Although the ADNI study set out to reduce the likelihood of including other pathologies such as vascular dementia, post mortem studies report that 2.4% of clinically diagnosed AD cases have isolated vascular pathology, and approximately one-third may have vascular pathology in addition to AD (Jellinger, 2006). The proportion of MCI and control subjects with preclinical or prodromal vascular dementia or mixed dementia may be higher. In ADNI, only approximately 60% of subjects had CSF taken at baseline, limiting the group sizes and therefore the power to detect potential relationships between CSF variables and brain volume losses in this study. A major limitation was that the ADNI study did not acquire fluid attenuated inversion recovery (FLAIR) images, which can improve the accuracy of WMH volume estimation. Such acquisitions have been included in ADNI2. Although our results are potentially useful for the planning of future studies, the generalizability of the findings may be limited to those studies with recruitment similar to that of ADNI, as relationships between CSF biomarkers, white matter disease, and brain atrophy rates may differ according to the study population characteristics. Finally, we did not investigate other forms of vascular pathology measurable on MRI, including lacunes and microbleeds. Microbleeds in particular could also help to distinguish WMH attributable to amyloid angiopathy rather than conventional vascular disease; but assessment of these requires T2*-weighted MRI, which is not available on the first ADNI dataset but is for ADNI 2 and ADNI GO. However, the independent association between WMH and CSF Aβ level found in our study argues against the majority of the observed WMH being related to amyloid angiopathy.

In conclusion, these data further support the notion that vascular damage is associated with brain volume loss in the context of AD pathology, predominantly in pre-dementia stages. In contrast, Aβ levels are also related to progressive cerebral volume loss in the MCI group, which comprises subjects with prodromal AD and those who will not progress to AD. We found evidence of an association between tau levels and atrophy only in the MCI group. Our findings are particularly relevant to the current interest in prevention trials (Mullard, 2012; Reiman et al., 2010; Richard et al., 2012; Selkoe, 2012); intervention to reduce vascular burden and its effects on progressive brain loss may be most effective early in dementia before symptoms become apparent. Furthermore, the finding that WMH explains significant additional variability beyond that explained by CSF measures suggests that WMH volume should be considered for stratification or adjustment to increase power in prevention trials.

## Disclosure statement

Josephine Barnes: Dr Barnes has received honoraria for reviewing grants for the Fundação para a Ciência e a Tecnologia-Portugal and was supported by an Alzheimer's Research UK fellowship.

Gerard R Ridgway: Dr Ridgway serves as an editorial board member for NeuroImage and has received honoraria for teaching on SPM courses. Jonathan M Schott: Dr Schott is a UK HEFCE Senior Lecturer and receives grant support from Alzheimer's Research UK Martin N Rossor: Professor Martin Rossor sits on the Data Monitoring Committee for Servier DMC Phase 2B AD Study S38093, and also sits on the Bapineuzumab Independent Safety Monitoring Committee for Janssen Al/Pfizer. Charlie DeCarli: Dr DeCarli is editor-in-Chief of ADAD. He has received honoraria for speaking at various academic centers and teaching at the AAN and has consulted with Avid and Takeda over the last year. Nick C Fox: Dr Fox has served on the scientific advisory boards of Alzheimer's Research Forum, Alzheimer's Society and Alzheimer's Research Trust and editorial boards of Alzheimer's Disease and Associated Disorders, Neurodegenerative Diseases, and Alzheimer's Research and Therapy. He holds a patent for QA Box that may accrue revenue. In the last 5 years his research group has received payment for consultancy or for conducting studies from Abbott Laboratories, AstraZeneca, AVID, Bristol-Myers Squibb, Elan Pharmaceuticals, Eisai, Eli Lilly, GE Healthcare, IXICO, Janssen (JAI), Lundbeck, Neurochem Inc, Pfizer Inc, Sanofi-Aventis, Teva and Wyeth Pharmaceuticals. He receives research support from MRC [G0801306 (PI), G0601846 (PI)] NIH [U01 AG024904 (Co-investigator(sub contract)], Alzheimer Research Trust [ART/RF/2007/1 (PI)] and NIHR (Senior Investigator). None of the other authors have any conflicts of interest to declare.

## Figures and Tables

**Table 1:** Subject demographics, genetics, vascular risk factors, and volumetric imaging summary statistics

	Controls, n = 197	MCI, n = 331	AD, n = 146	*p* value across 3 groups
Age, y	76.0 (5.1) [75.2, 76.7]	74.8 (7.2) [74.1, 75.6]	75.3 (7.3) [74.1, 76.5]	0.18
Gender, n (%) female	93 (47)	122 (37)	69 (47)	0.025
MMSE/30	29.1 (1.0) [29.0, 29.2]	27.0 (1.8) [26.8, 27.2]	23.4 (1.9) [23.1, 23.7]	<0.001
Diastolic BP, mm Hg	74.7 (10.5) [73.2, 76.2]	74.6 (9.5) [73.5, 75.6]	74.6 (9.3) [73.1, 76.2]	0.99
Systolic BP, mm Hg	134.9 (16.6) [132.6, 137.3]	135.4 (18.6) [133.4, 137.4]	136.5 (17.0) [133.7, 139.3]	0.71
Diabetes, n (%) history	10 (5)	26 (8)	8 (5)	0.46
Smoking, n (%)				
Never	122 (62)	199 (60)	93 (64)	0.97
Previous	69 (35)	121 (37)	49 (33)	
Present	6 (3)	11 (3)	4 (3)	
*APOE* ε4, n (%)				
0 alleles	140 (71)	152 (46)	48 (33)	<0.001
1 allele	52 (26)	139 (42)	68 (47)	
2 alleles	5 (3)	40 (12)	30 (21)	
WMH volume, mL				
Median (IQR)	0.25 (0.48)	0.25 (0.52)	0.36 (1.00)	0.002^c^
Brain volume/TIV	0.69 (0.04) [0.68, 0.69]	0.67 (0.04) [0.67, 0.68]	0.66 (0.04) [0.65, 0.66]	<0.001
Interval, days	395.8 (25.7) [392.2, 399.5]	394.0 (24.6) [391.3, 396.6]	392.5 (23.3) [388.7, 396.3]	0.45
BSI, mL/year	5.92 (6.08) [5.06, 6.77]	10.65 (8.23) [9.76, 11.54]	14.09 (8.00) [12.78, 15.40]	<0.001
CSF Aβ1-42^a^, pg/ml	204 (55) [193, 215]	163 (54) [155, 171]	141 (40) [132, 150]	<0.001
CSF tau^b^, pg/ml	70 (28) [64, 75]	100 (51) [92, 108]	124 (57) [112,137]	<0.001

Values reported are mean (SD) [95% CI] unless otherwise specified. Key: BP, blood pressure; BSI, boundary shift integral; CSF, cerebrospinal fluid; IQR, interquartile range; MMSE, Mini Mental State Examination; TIV, total intracranial volume; WMH, white matter hyperintensity.

a Available in 101 control, 168 MCI, and 82 AD subjects.

b Available in 101 control, 165 MCI, and 80 AD subjects.

c *p* value from comparison across groups of WMH on log scale.

**Table 2:** Adjusted regression coefficients [95% confidence intervals], *p* values, and semi-partial r^2^ values for associations with brain atrophy (BSI, mL/y)

	Controls	MCI subjects	AD subjects
Model 1	n = 197	n = 331	n = 146
WMH (doubling)	0.46 [0.09, 0.83] *p* = 0.015 r^2^ = 0.030	0.08 [−0.29, 0.45] *p* = 0.67 r^2^ ≤ 0.001	0.15 [−0.44, 0.73] *p* = 0.62 r^2^ = 0.002
Model 2	n = 101	n = 168	n = 82
WMH (doubling)	0.78 [0.28, 1.28] *p* = 0.003 r^2^ = 0.080	0.07 [−0.45, 0.59] *p* = 0.80 r^2^ ≤ 0.001	0.04 [−0.63, 0.72] *p* = 0.90 r^2^ ≤ 0.001
Aβ1-42 (per 10 pg/mL)	−0.36 [−0.57, −0.14] *p* = 0.001 r^2^ = 0.091	−0.43 [−0.66, −0.20] *p* < 0.001 r^2^ = 0.075	−0.28 [−0.68, 0.12] *p* = 0.17 r^2^ = 0.024
Model 3	n = 101	n = 165	n = 80
WMH (doubling)	0.82 [0.29, 1.34] *p* = 0.003 r^2^ = 0.087	0.22 [−0.33, 0.77] *p* = 0.43 r^2^ = 0.004	0.05 [−0.64, 0.75] *p* = 0.88 r^2^ ≤ 0.001
tau (per 10 pg/mL)	0.17 [−0.27, 0.62] *p* = 0.44 r^2^ = 0.006	0.28 [0.02, 0.54] *p* = 0.04 r^2^ = 0.027	0.02 [−0.28, 0.32] *p* = 0.89 r^2^ ≤ 0.001
Model 4	n = 101	n = 165	n = 80
WMH (doubling)	0.78 [0.28, 1.29] *p* = 0.003 r^2^ = 0.080	0.14 [−0.40, 0.68] *p* = 0.60 r^2^ = 0.002	0.04 [−0.64, 0.73] *p* = 0.90 r^2^ ≤ 0.001
Aβ1-42 (per 10 pg/mL)	−0.36 [−0.58, −0.14] *p* = 0.002 r^2^ = 0.086	−0.40 [−0.65, −0.14] *p* = 0.002 r^2^ = 0.054	−0.30 [−0.72, 0.11] *p* = 0.14 r^2^ = 0.028
tau (per 10 pg/mL)	<0.01 [−0.44, 0.44] *p* > 0.99 r^2^ ≤ 0.001	0.11 [−0.16, 0.39] *p* = 0.41 r^2^ = 0.004	−0.01 [−0.31, 0.30] *p* = 0.97 r^2^ ≤ 0.001

Key: AD, Alzheimer's disease; BSI, boundary shift integral; MCI, mild cognitive impairment; WMH, white matter hyperintensity. Model 1: Association between WMH and BSI, adjusting for head size. Model 2: Adjusted association of WMH and Aβ1-42 with BSI. Model 3: Adjusted association of WMH and tau with BSI. Model 4: Adjusted association of WMH, Aβ1-42 and tau with BSI.
