# Supplementary material for: Vascular and Alzheimer's disease markers independently predict brain atrophy rate in Alzheimer's Disease Neuroimaging Initiative controls
Source: Neurobiol Aging. 2013 Aug;34(8):1996–2002. doi: 10.1016/j.neurobiolaging.2013.02.003 (PMC3810644; doi:10.1016/j.neurobiolaging.2013.02.003)
Supplement: Supplementary Tables 1 and 2 [file mmc1.docx]

|  | Controls | MCI | AD |
| --- | --- | --- | --- |
| Model 1  WMH (doubling) | n=197  0.47 [0.10, 0.84]  p=0.014  r^2^=0.031 | n=331  0.08 [-0.31, 0.46]  p=0.70  r^2^≤0.001 | n=146  0.20 [-0.40, 0.80]  p=0.52  r^2^=0.003 |
| Model 2  WMH (doubling)  Aβ1-42 (per 10 pg/ml) | n=101  0.83 [0.33, 1.33]  p=0.001  r^2^=0.089  -0.33 [-0.55, -0.11]  p=0.003  r^2^=0.076 | n =168  <0.01 [-0.57, 0.57]  p=0.99  r^2^≤0.001    -0.44 [-0.67, -0.20]  p<0.001  r^2^=0.076 | n=82  0.12 [-0.58, 0.82]  p=0.73  r^2^=0.002    -0.26 [-0.67, 0.14]  p=0.20  r^2^=0.021 |
| Model 3  WMH (doubling)  tau (per 10 pg/ml) | n=101  0.88 [0.36, 1.40]  p=0.001  r^2^=0.099  0.20 [-0.24, 0.64]  p=0.37  r^2^=0.007 | n=165  0.22 [-0.38, 0.82]  p=0.47  r^2^=0.003  0.28 [0.02, 0.54]  p=0.04  r^2^=0.027 | n=80  0.13 [-0.58, 0.85]  p=0.71  r^2^=0.002    0.02 [-0.28, 0.32]  p=0.90  r^2^≤0.001 |
| Model 4  WMH (doubling)  Aβ1-42 (per 10 pg/ml)  tau (per 10 pg/ml) | n=101  0.83 [0.33, 1.33] p=0.001  r^2^=0.089  -0.33 [-0.55, -0.10]  p=0.005  r^2^=0.069  0.04 [-0.40, 0.48] p=0.87  r^2^≤0.001 | n=165  0.09 [-0.50, 0.68]  p=0.77  r^2^≤0.001  -0.41 [-0.66, -0.15] p=0.002  r^2^= 0.055  0.11 [-0.17, 0.39]  p=0.44  r^2^= 0.003 | n=80  0.11 [-0.60, 0.82] p=0.76  r^2^≤0.001  -0.29 [-0.70, 0.12] p=0.17  r^2^= 0.025  -0.01 [-0.31, 0.30] p=0.96  r^2^≤0.001 |

**Supplementary table 1. Adjusted regression coefficients [95% confidence intervals], p values and semi-partial r^2^ values for associations with brain atrophy (BSI, ml/year) with adjustment for baseline brain volume.**

(1): association between WMH and BSI, adjusting for head size and baseline brain volume.

(2): adjusted association of WMH and Aβ1-42 with BSI.

(3): adjusted association of WMH and tau with BSI.

(4): adjusted association of WMH, Aβ1-42 and tau with BSI.

|  | Controls | MCI | AD |
| --- | --- | --- | --- |
| Model 1  WMH (doubling) | n = 197  0.44 [0.06, 0.82] p=0.023  r^2^= 0.026 | n=331  0.21 [-0.18, 0.59]  p=0.29  r^2^= 0.003 | n=146  0.33 [-0.25, 0.92]  p=0.26  r^2^= 0.008 |
| Model 2  WMH (doubling)  Aβ1-42 (per 10 pg/ml) | n=101  0.80 [0.29, 1.32] p=0.002  r^2^= 0.081  -0.36 [-0.58, -0.15] p=0.001  r^2^= 0.092 | n=168  0.23 [-0.31, 0.77]  p=0.40  r^2^= 0.004  -0.41 [-0.64, -0.18]  p=0.001  r^2^= 0.068 | n=82  0.18 [-0.50, 0.86]  p=0.60  r^2^= 0.003  -0.19 [-0.60, 0.21]  p=0.35  r^2^= 0.011 |
| Model 3  WMH (doubling)  tau (per 10 pg/ml) | n=101  0.84 [0.30, 1.37]  p=0.003  r^2^= 0.088  0.19 [-0.27, 0.65]  p=0.41  r^2^= 0.006 | n=165  0.40 [-0.17, 0.97]  p=0.17  r^2^= 0.011  0.28 [0.03, 0.54]  p=0.032  r^2^= 0.028 | n=80  0.21 [-0.49, 0.90]  p=0.55  r^2^= 0.004  -0.02 [-0.32, 0.28]  p=0.90  r^2^≤ 0.001 |
| Model 4  WMH (doubling)  Aβ1-42 (per 10 pg/ml)  tau (per 10 pg/ml) | n=101  0.80 [0.29, 1.32]  p=0.003  r^2^= 0.081  -0.36 [-0.58, -0.14]  p=0.002  r^2^= 0.086  0.02 [-0.43, 0.47]  p=0.94  r^2^≤ 0.001 | n=165  0.31 [-0.25, 0.86]  p=0.28  r^2^= 0.007  -0.38 [-0.63, -0.12]  p=0.004  r^2^= 0.048  0.13 [-0.15, 0.40]  p=0.37  r^2^=0.005 | n=80  0.18 [-0.52, 0.88]  p=0.61  r^2^= 0.003  -0.22 [-0.64, 0.19]  p=0.29  r^2^= 0.014  -0.03 [-0.33, 0.27]  p=0.82  r^2^≤ 0.001 |

**Supplementary table 2. Adjusted regression coefficients [95% confidence intervals], p values and semi-partial r^2^ values for associations with brain atrophy (BSI, ml/year) with adjustment for age.**

(1): association between WMH and BSI, adjusting for head size and age.

(2): adjusted association of WMH and Aβ1-42 with BSI.

(3): adjusted association of WMH and tau with BSI.

(4): adjusted association of WMH, Aβ1-42 and tau with BSI.
